# A case report of hyponatremia induced by hypopituitarism following severe traumatic brain injury and literature review

**DOI:** 10.3389/fmed.2026.1847538

**Published:** 2026-05-29

**Authors:** Dan-Dan Liu, Shan-Shan Xu, Xu An, Yue-Fu Wang, Jian-Xin Zhou

**Affiliations:** 1 Department of Surgical Intensive Care Unit, Emergency and Critical Care Medical Center, Beijing Shijitan Hospital, Capital Medical University, Beijing, China; 2 Emergency and Critical Care Medical Center, Beijing Shijitan Hospital, Capital Medical University, Beijing, China

**Keywords:** cerebral salt wasting syndrome, hyponatremia, hypopituitarism, inappropriate antidiuretic hormone secretion, neurocritical care

## Abstract

Hyponatremia induced by hypopituitarism is an underrecognized complication in neurocritical care settings. This article presents a case of a patient with severe traumatic brain injury who developed euvolemic hyponatremia one month after the injury, accompanied by decreased serum osmolality. The hyponatremia remained refractory to conventional management, including fluid restriction and hypertonic saline supplementation. Subsequent endocrine evaluation confirmed hypopituitarism, characterized by markedly suppressed ACTH (<1 pg/ml) and low morning cortisol (1.68 μg/dl). Notably, serum sodium levels normalized following the initiation of exogenous hormone replacement. Our findings may represent an important but underrecognized cause of hyponatremia in neurocritical care patients, particularly those with severe acute brain injuries. Comprehensive testing for neuroendocrine hormones in this subset of patients is beneficial.

## Introduction

Hyponatremia is prevalent among hospitalized patients with an incidence exceeding 15%, and reaching up to 30% in various acute and chronic disease populations (1). In neurocritical care units, the incidence ranges from 9.6% to 51% (2, 3). Beyond its prevalence, hyponatremia is a major factor contributing to the increased disease burden, associated with prolonged hospitalization (3.2 days), a 32% readmission rate, and an average cost escalation of $2,000 per case. Critically, each 1 mmol/L decline in serum sodium is correlated with a 2.3% increase in mortality. It is an independent risk factor for poor long-term outcomes in neurocritically ill patients (2, 4).

Hyponatremia in neurocritical care settings is primarily attributed to the Syndrome of Inappropriate Antidiuretic Hormone Secretion (SIADH) and Cerebral Salt Wasting Syndrome (CSWS). SIADH is characterized by the inappropriate elevation of antidiuretic hormone (ADH) levels relative to plasma osmolality, leading to excessive water retention and subsequent dilutional hyponatremia. CSWS is a clinical syndrome characterized by hyponatremia and hypovolemia, primarily caused by excessive renal sodium and secondary water wasting following central nervous system injury. The underlying mechanisms of CSWS involve the disruption of sympathetic nervous system regulation and the pathological release of natriuretic factors, which impair proximal tubule re-absorption of sodium, uric acid, and water, while also affecting renin release.

The hypothalamus-pituitary axis serves as the central regulator of water and sodium homeostasis. Dysregulation of this axis, often manifesting as hormonal fluctuations following acute brain injury (ABI), is a frequent cause of hyponatremia. Within this regulatory pathway, hyponatremia caused by secondary adrenal insufficiency-a consequence of anterior pituitary hypofunction-is often overlooked in clinical practice. This article presents a case of hyponatremia induced by anterior hypopituitarism following severe TBI, aiming to enhance clinicians' diagnostic and therapeutic precision when managing hyponatremia in neurocritical care patients.

## Case report

### Chief complaint

A 26-year-old male presented to the neuro-ICU on July 26, 2018, with a 1-hour history of unconsciousness resulting from a motor vehicle collision (MVC).

### Physical examination (admission)

On admission, the patient's vital signs were: temp 36.2 °C, HR 61 bpm, BP 100/78 mmHg, and SpO2 100%. Neurological assessment showed deep coma with a Glasgow Coma Scale (GCS) score of 2T (E1VTM2). Pupils were bilaterally dilated to 5 mm and non-reactive to light. corneal reflex (–), cough reflex (+). Mechanical ventilation was administered via endotracheal intubation, with the presence of spontaneous respiration. Motor examination revealed symmetrical flaccid paralysis with diminished muscle tone and positive bilateral Babinski signs.

### Past medical history

Splenectomy 3 years prior (post-motor vehicle accident). No history of cardiovascular or endocrine disorders and no known food or drug allergies.

### Imaging and diagnosis

Initial cranial computed tomography (CT) revealed brainstem injury, diffuse cerebral edema, bilateral frontotemporal subdural hematomas, subarachnoid hemorrhage (SAH), and bilateral frontotemporal contusions, accompanied by bilateral pulmonary contusion.

### Initial management

Due to the severity of the injuries, no surgery was performed. Management focused on a conservative multimodal approach, including: dehydration therapy with mannitol for intracranial pressure (ICP) control; sedation and analgesia to minimize cerebral metabolic demand; protective mechanical ventilation; administration of vasopressin (pituitrin) and desmopressin (DDAVP) to manage diabetes insipidus and correct hypernatremia.

### Development of refractory hyponatremia

One month post-TBI, the patient developed persistent hyponatremia, with serum sodium levels fluctuating between 120 and 130 mmol/L. A low dose of DDAVP was taken because of the persistent diabetes insipidus. The profound hyponatremia persisted even after the discontinuation of DDAVP therapy for more than 24 hours, exceeding the expected duration of action for antidiuretic effects. Other clinical assessment indicated euvolemia (normal skin turgor, moist mucous membranes, and an absence of peripheral edema), characterized by stable hemodynamics, a hematocrit (Hct) of 38%, and the absence of signs of dehydration (e.g., sunken orbits or dry skin). The calculated serum osmolality was approximately 265 mOsm/L, while renal function remained normal (creatinine 76 umol/L). CSWS was considered unlikely based on the patient's euvolemic status, and the patient was managed under a presumptive diagnosis of SIADH. However, conventional treatments, including fluid restriction (limit to 1,000 ml/day) and hypertonic saline supplementation, yielded poor results.

### Endocrine evaluation and diagnosis

When the initial measures proved refractory (with serum sodium remaining at 120–130 mmol/L), a neuroendocrine evaluation became the diagnostic priority. Subsequent endocrine screening revealed profound deficiencies: adrenocorticotropic hormone (ACTH) < 1 pg/ml (Ref: 7.2–63.3 pg/ml) and 8:00 AM cortisol of 1.68 ug/dl (Ref: 8.7–22.4 pg/ml). Following a consultation with the endocrinology department, the patient was diagnosed with hypopituitarism and secondary adrenal insufficiency.

### Treatment and follow-up

Glucocorticoid replacement was initiated with cortisone acetate. Upon observing that standard replacement doses failed to resolve the electrolyte imbalance, the dosage was meticulously titrated in response to the patient's refractory hyponatremia and hemodynamic status. The regimen was eventually escalated to a total daily dose of 75 mg, administered in a divided schedule (37.5 mg at 8:00 AM, 25 mg at 12:00 PM, and 12.5 mg at 6:00 PM), which achieved and maintained the normalization of serum sodium levels. At the 3-month follow-up, although ACTH remained suppressed (< 1 pg/ml), the cortisol rhythm showed improvement (8:00 AM: 14.37 ug/dl; 4:00 PM: 6.86 ug/dl; 12:00 AM: 3.85 ug/dl), and normalnatremia was maintained. Additionally, Levothyroxine (125 ug/day) was administered to address concurrent central hypothyroidism. Due to technical limitations and the patient's condition, Magnetic Resonance Imaging (MRI) was not performed; cranial CT provided limited visualization of the pituitary region (Figure 1).

**Figure 1:**
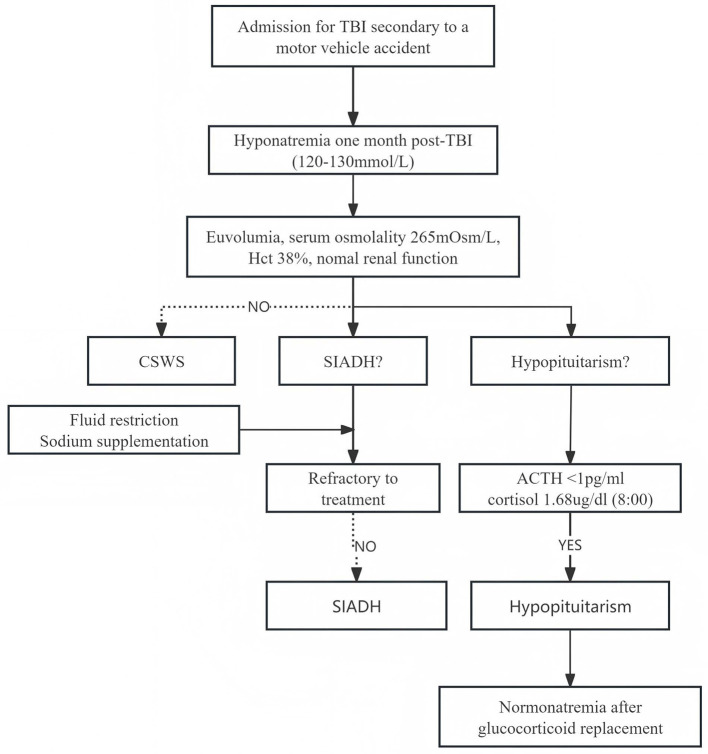
Diagnostic and therapeutic flowchart for post-TBI hyponatremia.

Four months post-TBI, an attempt was made to taper the cortisone acetate dosage. However, this resulted in a recurrence of hyponatremia. Upon reverting to the previous replacement regimen, the patient's serum sodium levels stabilized once again.

At this stage, the patient remained in a persistent coma with a GCS score of 4 (E2VTM2), and was maintained on mechanical ventilation via a tracheostomy tube. Although spontaneous respiratory triggering was observed, weaning trials were unsuccessful as arterial blood gas analysis failed to meet extubation criteria. Neurological examination revealed a decerebrate state. Pupils were bilaterally equal at 3.5 mm with sluggish light reflexes.

Functional magnetic resonance imaging (fMRI) was performed using an MR-compatible ventilator to routinely evaluate the patient's neurological function.

The results revealed severe structural damage to the hypothalamus-pituitary axis (Figure 2). Arterial Spin Labeling (ASL) demonstrated residual cerebral blood flow signals at the skull base, and Diffusion Tensor Imaging (DTI) demonstrated profound disruption of multiple white matter tracts, including the corpus callosum and cingulate gyrus (Figure 3).

**Figure 2:**
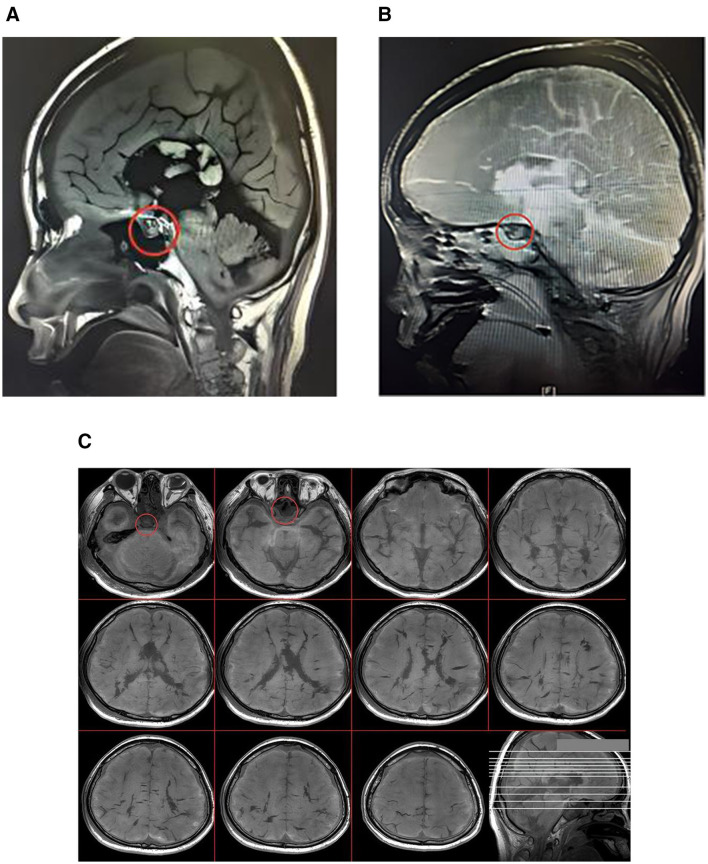
Cranial MRI imaging revealing severe pituitary gland injury. **(A)** Sagittal T1-weighted sequence shows significant volume reduction and signal intensity alterations in the Sella Turcica region, consistent with severe pituitary atrophy and necrosis. **(B)**. Sagittal T2-weighted sequence further delineates the destruction of the pituitary parenchyma, suggesting tissue rarefaction within the pituitary gland. **(C)** Axial T1-weighted sequence provides a cross-sectional perspective of the pituitary fossa, demonstrating diffuse atrophy and a marked absence of the normal distinct borders of the pituitary stalk and neurohypophysis.

**Figure 3:**
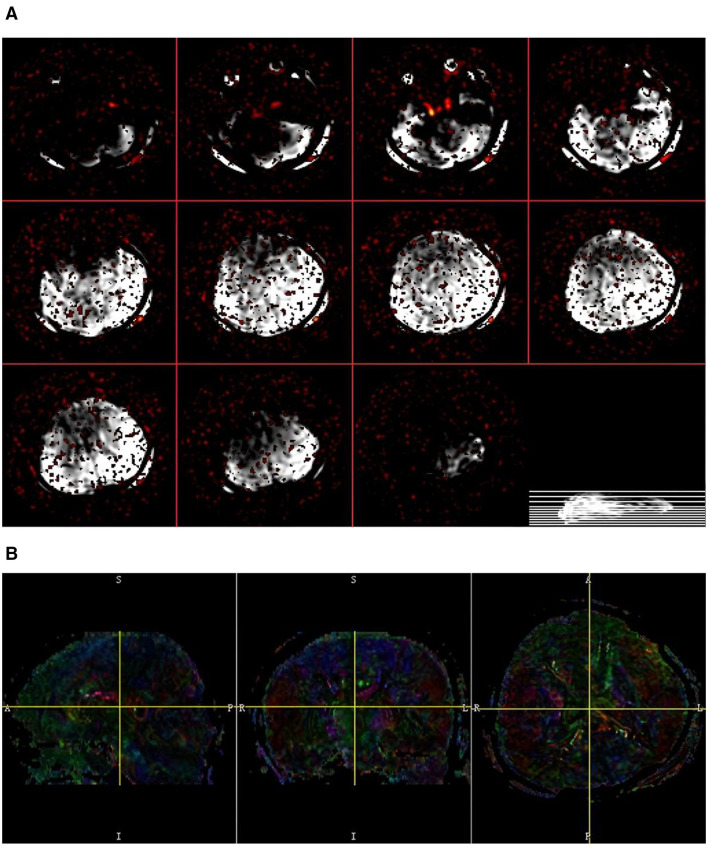
Functional neuroimaging via ASL and DTI sequences. **(A)** Arterial spin labeling (ASL) sequence reveals a critical reduction in cerebral blood flow. Only the major vessels at the skull base remain visible (residual flow), while the supra-tentorial parenchymal perfusion is severely attenuated, indicating a diffuse state of hypoperfusion. **(B)** Diffusion tensor imaging (DTI) illustrates catastrophic disruption of major white matter pathways. There is a marked loss of fractional anisotropy (FA) and physical discontinuity within the corpus callosum and the cingulate gyrus bundles. These findings are consistent with the clinical diagnosis of a prolonged disorder of consciousness (pDOC) secondary to diffuse axonal injury.

### Clinical status and final outcome

Following multidisciplinary evaluation by neurology and neurosurgery departments, the patient did not meet the criteria for brain death. Instead, he was diagnosed with a prolonged disorder of consciousness (pDOC) in a chronic coma. Despite intensive supportive care, the clinical course was complicated by recurrent refractory infections and septic shock. The patient passed away on March 9, 2019. Timeline of relevant events in the patient history is summarized in Figure 4.

**Figure 4:**
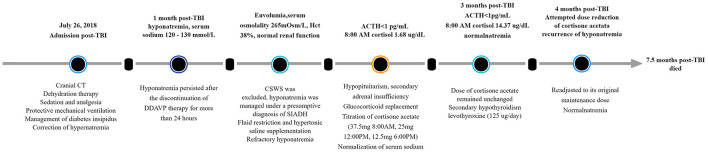
Timeline of relevant events in the patient history.

## Discussion

This report describes a case of hyponatremia secondary to hypopituitarism caused by severe hypothalamic-pituitary injury. The patient presented with euvolemic hyponatremia, with serum sodium levels fluctuating between 120 and 130 mmol/L and calculated serum osmolality below normal. After excluding CSWS, conventional SIADH treatments, including fluid restriction and sodium supplementation, were found to be ineffective. Subsequent laboratory tests revealed ACTH and cortisol levels below the reference ranges. Following the diagnosis of hypopituitarism and the initiation of exogenous hormone replacement therapy, serum sodium levels normalized.

Despite significant advancements in neurocritical care over the past decades, the diagnostic approach to hyponatremia remains susceptible to long-standing pitfalls. This case underscores a persistent clinical oversight: the pre-mature diagnosis of SIADH without the systematic exclusion of secondary adrenal insufficiency. Although the prerequisite for endocrine screening was established as early as 1967, suboptimal adherence to this criterion remains remarkably prevalent in modern neuro-ICUs. We contend that revisiting this foundational standard is more critical now than ever. By utilizing this clinical encounter as a central vehicle and integrating a comprehensive literature review dating back to 1967, this work serves as a pedagogical model for the differential diagnosis of hyponatremia. Rather than a mere anecdotal report, it highlights an enduring diagnostic dilemma that continues to compromise patient care and clinical outcomes.

The regulation of water and sodium balance is a tightly integrated physiological process: fluctuations in blood volume directly modulate sodium concentration, while shifts in serum sodium levels, in turn, redistribute body fluids. The hypothalamic-pituitary axis serves as the central command for this homeostasis, primarily through the secretion of corticotropin-releasing hormone (CRH) and arginine vasopressin (AVP), also known as ADH. CRH stimulates the adenohypophysis (anterior pituitary) to secrete ACTH, which subsequently triggers the adrenal cortex to release cortisol. Cortisol facilitates renal free water excretion partly by modulating renal hemodynamics and osmotic gradients in the renal collecting ducts. Crucially, cortisol exerts an inhibitory effect on AVP secretion. In states of hypocortisolism (such as anterior hypopituitarism), this inhibitory tone is lost, leading to a relative excess of AVP. This results in pathological water retention and the development of dilutional hyponatremia (5). Synthesized in the hypothalamus and released via the neurohypophysis (posterior pituitary), AVP binds to V2 receptors on the basolateral membrane of collecting duct principle cells, increasing water permeability and re-absorption. Furthermore, AVP enhances NaCl re-absorption in the thick ascending limb of the loop of Henle and increases urea permeability in the collecting duct, thereby strengthening the medullary osmotic gradient. Its vasoconstrictive effect on the vasa recta further reduces medullary blood flow, thereby promoting maximal water reabsorption (5, 6). In addition to the hypothalamic-pituitary axis, the renin-angiotensin-aldosterone system (RAAS) and atrial natriuretic peptide (ANP) act as critical auxiliary regulators in maintaining systemic fluid and electrolyte balance.

Hyponatremia in neurocritical care units is pre-dominantly attributed to SIADH and CSWS. Since the first case described by Schwartz in 1957 (7) and the establishment of diagnostic criteria in 1967 (8), SIADH has been recognized as the leading cause of hyponatremia, accounting for 35%−40% of all inpatient cases and approximately 50% within neurocritical care settings (9). SIADH typically manifests as euvolemic hyponatremia, with no clear clinical evidence of hypovolemia or hypervolemia. Diagnostic criteria are as follows: serum osmolality: < 275 mOsm/kg, urine osmolality: >100 mOsm/kg, urinary sodium: >30 mmol/L (under normal salt and water intake). Exclusionary criteria: normal renal function, no diuretic use, and the absence of other causes for euvolemic hyponatremia (e.g., glucocorticoid deficiency or severe hypothyroidism) (8). Management prioritizes fluid restriction, which is cost-effective but often limited by poor patient compliance and the slow rate of sodium correction. Secondary treatments include oral salt supplementation and AVP receptor antagonists, such as tolvaptan (10). CSWS was first reported by Peters et al. in 1950 (11), presents as hypovolemic hyponatremia. Patients typically exhibit signs of dehydration, such as hypotension, decreased skin turgor, or elevated hematocrit. Biochemical findings include decreased serum osmolality, elevated urine osmolality (usually >300 mOsm/kg), and high urinary sodium (>40 mmol/L). A characteristic feature is hypouricemia resulting from excessive renal urate wasting. While CSWS is often self-limiting within 3–4 weeks, its primary management requires aggressive fluid and sodium replacement.

Both SIADH and CSWS are characterized by a pathological excess or overactivity of regulatory hormones. SIADH involves inappropriately elevated levels of AVP, while CSWS is driven by the potentiation of natriuretic peptides and sympathetic dysregulation. In contrast, our patient exhibited a profound hormonal deficiency, specifically within the hypothalamic-pituitary-adrenal (HPA) axis. The primary lesion in SIADH typically involves the neurohypophysis (posterior pituitary) or its hypothalamic regulation. CSWS involves a complex interplay between renal afferent nerves, the sympathetic nervous system, and systemic natriuretic factors. Conversely, the localized injury in this case was centered on the adenohypophysis (anterior pituitary).

In clinical practice, hyponatremia secondary to hypopituitarism presents as a classic euvolemic hypoosmolar state, making it phenotypically indistinguishable from SIADH. This similarity creates a significant diagnostic challenge, particularly because the direct measurement of AVP is plagued by its short half-life and pre-analytical instability, rendering accurate clinical assessment difficult. Consequently, clinicians often rely on exclusionary criteria, where the evaluation of the HPA axis becomes paramount. Hypopituitarism is frequently secondary to severe neurological injuries, including SAH, skull base meningitis, brain abscess, TBI, empty sella syndrome, brain tumors, and pituitary apoplexy. Epidemiological data underscore the prevalence of this “hidden” etiology: in SAH populations, research indicates that among patients with euvolemic hyponatremia, approximately 8.2% are attributed to ACTH deficiency rather than primary SIADH (12). In general medical settings, studies have reported that the incidence of anterior hypopituitarism among hyponatremic inpatients can reach as high as 11.1% (13).

The mechanism by which hypopituitarism induces hyponatremia are multifatorial, involving both direct renal effects and systemic hormonal dysregulation. 1) Impaired free water clearance: a reduction in circulating cortisol directly impairs the kidneys' capacity to excrete free water. This leads to pathological water retention and the development of dilutional hyponatremia. 2) Disruption of the cortisol-AVP feedback loop: under physiological conditions, cortisol exerts a potent inhibitory effect on the secretion of AVP. In the setting of hypocortisolism, this negative feedback is lost, and the stimulatory drive for ACTH production simultaneously wanes. Consequently, AVP levels become inappropriately elevated even in the absence of a decline in plasma osmolality (5). This state of “non-osmotic AVP release” effectively mimics the biochemical profile of SIADH, yet remains fundamentally driven by an adrenal insufficiency that can only be corrected through targeted glucocorticoid replacement.

In the diagnostic evaluation of hypopituitarism, while the assessment of thyroid axis deficiency is a standardized procedural requirement, hyponatremia in patients with concurrent hypothyroidism and hypocortisolism is primarily driven by glucocorticoid deficiency. Definitive diagnosis relies on stringent biochemical markers: serum Cortisol: a morning serum cortisol level < 5 ug/dl is diagnostic of adrenal insufficiency. ACTH Levels: plasma ACTH concentrations that are suppressed or at the lower limit of the reference range (typically < 20 pg/ml) further support a secondary (central) etiology. For patients with indeterminate morning cortisol levels (between 5 and 10 ug/dl), dynamic testing is necessitated. The CRH stimulation test remains a primary diagnostic tool (14, 15). Although the Insulin Tolerance Test (ITT) is considered an alternative gold standard (14), it carries significant clinical risks—including the induction of seizures and exacerbation of ischemic heart disease—as it requires achieving a blood glucose level < 2.2 mmol/L. Consequently, the ITT should only be performed after the initial correction of hyponatremia to ensure patient safety.

For patients with ACTH deficiency secondary to pituitary dysfunction, glucocorticoid replacement is the cornerstone of therapy. Standard pharmacological options include oral hydrocortisone or cortisone acetate. The latter exhibits a delayed onset of action, as it requires hepatic conversion to hydrocortisone via 11β-hydroxysteroid dehydrogenase (11βHSD) type 1. In clinical practice, hydrocortisone remains the preferred agent for adrenal insufficiency in most countries. Normal functioning adrenal glands produce between 5 mg and 10 mg of cortisol per m^2^ body surface area in a day, which (allowing for incomplete intestinal absorption) is equivalent to an oral replacement dose of 15–25 mg hydrocortisone per day for an adult. Small and frequent dosing gives a more physiological plasma cortisol profile. Most adults take two or three doses of hydrocortisone daily, but some prefer four or even more. The first and largest dose should be taken as soon as the patient is awake, and the last dose should be taken 4–6 h before bedtime to avoid sleep disturbances. Evening hydrocortisone dosing has been associated with insulin resistance and should be avoided. The increase in area under the curve and maximum serum concentrations of cortisol with increasing doses is linear but not proportional. Thus, there is little advantage in taking a morning hydrocortisone dose greater than 10 mg because blood cortisol concentrations are not substantially increased by taking single doses higher than this (14, 15). Notably, the corticosteroid requirements in this case exceeded standard physiological replacement levels. The recurrence of hyponatremia during subsequent tapering attempts highlights the imperative for individualized dose titration guided by clinical and biochemical responses, rather than a rigid adherence to generalized protocols.

It must be emphasized that the diagnosis in this patient was established strictly through biochemical assessment—characterized by refractory hyponatremia and suppressed cortisol/ACTH levels—rather than neuroimaging findings. The MRI was utilized to investigate the pathophysiological substrates of pituitary dysfunction and did not serve as a diagnostic pre-requisite. These advanced sequences (DTI and ASL) were performed as part of a comprehensive institutional research protocol for severe traumatic brain injury (sTBI) to explore microstructural integrity and perfusion status. They are not mandatory for routine clinical management; furthermore, a normal MRI appearance does not preclude a diagnosis of hypopituitarism, for which biochemical screening remains the gold standard.

## Conclusion

Hyponatremia secondary to hypopituitarism is most commonly reported in the literature as isolated case reports (16–19). However, our clinical experience suggests that this etiology is far from rare. It remains an underrecognized complication across the entire spectrum of TBI, from mild to extremely severe cases. A diagnostic strategy including comprehensive neuroendocrine hormonal screening and advanced functional neuroimaging is essential for accurate differentiation. As demonstrated in this case, the timely identification of anterior pituitary dysfunction and the prompt initiation of hormone replacement therapy are critical. Correcting the underlying hormonal imbalance not only stabilizes serum sodium levels but also improves the clinical outcomes.

## Limitation

The urine sodium and urine osmolality values were not documented in this retrospective case. However, we believe the diagnosis of hypopituitarism and secondary adrenal insufficiency remains definitive based on the Clinical assessment, diagnostic workup, and therapeutic validation.

## Data Availability

The original contributions presented in the study are included in the article/supplementary material, further inquiries can be directed to the corresponding author.
